# Susceptibility of Clinical Enterobacterales Isolates With Common and Rare Carbapenemases to Mecillinam

**DOI:** 10.3389/fmicb.2020.627267

**Published:** 2021-01-12

**Authors:** Frieder Fuchs, Aysel Ahmadzada, Lars Plambeck, Thorsten Wille, Axel Hamprecht

**Affiliations:** ^1^Institute for Medical Microbiology, Immunology and Hygiene, Medical Faculty and University Hospital of Cologne, University of Cologne, Cologne, Germany; ^2^German Centre for Infection Research (DZIF), Partner Site Bonn-Cologne, Cologne, Germany; ^3^Institute for Medical Microbiology and Virology, University of Oldenburg, Oldenburg, Germany

**Keywords:** pivmecillinam, UTI, multidrug-resistant, OXA-48, IMI, NDM, CPE, amdinocillin

## Abstract

**Purpose**: To investigate the susceptibility of carbapenemase-producing Enterobacterales (CPE) to mecillinam based on the recently updated European Committee on Antimicrobial Susceptibility Testing (EUCAST) breakpoints for uncomplicated Urinary Tract Infection (uUTI).

**Methods**: The challenge collection consisted of 105 molecularly characterized Enterobacterales [*Klebsiella* spp. (*N* = 49), *Escherichia coli* (*N* = 30), *Enterobacter cloacae* (*n* = 13), *Citrobacter freundii* (*N* = 9), *Proteus mirabilis* (*N* = 3), and *Raoultella ornithinolytica* (*N* = 1)]. Isolates produced OXA-48 (*N* = 18), OXA-48-like (*N* = 18), VIM (*N* = 22), NDM (*N* = 22), KPC (*N* = 12), IMI (*N* = 9), IMP (*N* = 6), GES (*N* = 1), OXA-58 (*N* = 2) or combinations thereof (*N* = 5). MICs of carbapenems were determined by agar gradient diffusion (AGD). MICs of mecillinam were assessed by agar dilution (reference method) and compared to disk diffusion (DD) and AGD.

**Results**: Overall 23/105 CPE (21.9%) were susceptible to mecillinam. Susceptibility was observed in *E. coli* (*N* = 12), *E. cloacae* (*N* = 7), and *Klebsiella pneumoniae* (*N* = 4) producing IMI, OXA-48, OXA-48-like, and NDM-1 carbapenemases. MIC_50_ for mecillinam in all isolates was 128 mg/L while MIC_50_ for meropenem was 8 mg/L. Lower MICs for mecillinam were found in IMI (MIC_50_ 8 mg/L) and OXA-48-like (MIC_50_ 16 mg/L) producers. The comparison of the different susceptibility methods showed very major errors of 12.2% with AGD and 8.5% with disk diffusion when compared to the reference method.

**Conclusion**: Mecillinam susceptibility was restricted to isolates producing IMI-, OXA-48-like, and NDM-1 carbapenemases and was documented despite high carbapenem MICs in some isolates. Mecillinam could be a promising oral antimicrobial in uUTI caused by *E. coli* and *E. cloacae* isolates carrying IMI- and OXA-48-like carbapenemases; however, susceptibility testing by AGD and disk diffusion remains problematic.

## Introduction

Limited options for antimicrobial therapy in infections caused by carbapenemase-producing Enterobacterales (CPE) are a major global health concern with increasing morbidity and mortality (Nordmann and Poirel, 2014; Martin et al., 2018). In urinary tract infections (UTI) the prevalence of multidrug-resistant Enterobacterales is increasing in in- and outpatients (Mazzariol et al., 2017). In an observational study of over 20.000 community-onset culture-positive UTIs from the United States, carbapenem-resistant Enterobacterales (CRE) were found in 3.0% of cases (Zilberberg et al., 2017). Repurposing previously approved antimicrobials according to their potential in these difficult to treat infections is an important strategy for future disease management (Theuretzbacher et al., 2015).

Pivmecillinam is the oral prodrug of mecillinam, a beta-lactam antibiotic that has been known since the 1970s (Lund and Tybring, 1972; Neu, 1976). Its activity against multidrug-resistant Enterobacterales has led to first-line recommendations for the treatment of uncomplicated UTI (uUTI) in several guidelines (e.g., the updated German guidelines or the international clinical practice guidelines of Infectious Diseases Society of America (IDSA) and European Society of Clinical Microbiology and Infectious Diseases (ESCMID; Gupta et al., 2011; Kranz et al., 2017). The European Committee on Antimicrobial Susceptibility Testing (EUCAST) breakpoint is set at S ≤ 8 mg/L, R > 8 mg/L and can be applied to *Escherichia coli, Klebsiella* spp., *Proteus mirabilis*, *Enterobacter* spp., *Citrobacter* spp., and *Raoultella* spp. (EUCAST, 2020). CLSI has recently introduced a breakpoint for mecillinam, with S ≤ 8 mg/L, I = 16 mg/L, and R ≥ 32 mg/L, currently for *E. coli* only and not for other Enterobacterales (CLSI, 2020).

Most of the data on mecillinam susceptibility in multidrug-resistant Enterobacterales focus on *E. coli* and frequent resistance mechanisms, e.g., Extended-spectrum beta-lactamases (ESBL), AmpC, and some of the more common types of carbapenemases such as OXA-48 or NDM-1 (Perry et al., 2011; Marrs et al., 2014; Mutters et al., 2015; Samuelsen et al., 2017; Fuchs and Hamprecht, 2019a; Zykov et al., 2020).

The aim of the present study was to assess the *in vitro* activity of mecillinam in all species for which EUCAST breakpoints have been defined and in isolates producing frequent and rare carbapenemases.

## Materials and Methods

Isolates were selected from a previously described collection of molecularly characterized CPE from different clinical specimens (Gruber et al., 2015; Koroska et al., 2017; Hamprecht et al., 2018; Baeza et al., 2019; Fuchs and Hamprecht, 2019b; Greissl et al., 2019). Stratified by carbapenemase genes, isolates produced OXA-48 (*N* = 18), OXA-48-like (*N* = 18), VIM (*N* = 22), NDM (*N* = 22), KPC (*N* = 12), IMI (*N* = 9), IMP (*N* = 6), GES (*N* = 1), and OXA-58 (*N* = 2). Five isolates produced two carbapenemases, NDM + OXA-48-like (*N* = 3) and KPC + VIM (*N* = 2). Species included *Klebsiella pneumoniae* (*n* = 47), *E. coli* (*n* = 30), *Enterobacter cloacae* (*n* = 13), *Citrobacter freundii* (*n* = 9), *P. mirabilis* (*n* = 3), *Klebsiella oxytoca* (*n* = 2), and *Raoultella ornithinolytica* (*n* = 1). MICs of meropenem, imipenem, and ertapenem were determined by agar gradient diffusion (AGD) using MIC test strips (Liofilchem, Roseto degli Abruzzi, Italy). MICs of mecillinam were determined by the reference method agar dilution (mecillinam powder, Molekula, Munich, Germany) with Mueller-Hinton agar (Becton Dickinson, Sparks, MD, USA) according to EUCAST methodology (EUCAST, 2000). Disk diffusion with mecillinam disks (10 μg, Oxoid, Wesel, Germany) and AGD with mecillinam MIC test strips (Liofilchem) was additionally performed and compared to reference methodology results (Matuschek et al., 2014; EUCAST, 2018). In isolates with discrepant results (difference in MIC of >2 dilutions between reference method and AGD or categorical disagreement in any method) susceptibility testing was repeated. Two additional CREs (*K. pneumoniae* and *E. cloacae*) that were carbapenemase negative were used as comparators; *E. coli* ATCC 25922 served as quality control strain. The EUCAST susceptibility breakpoint of ≤8 mg/L for uUTI was used for interpretation.

## Results

### Mecillinam and Carbapenem Susceptibility

Overall 23/105 (21.9%) of isolates were susceptible to mecillinam. For carbapenems, MIC_50/90_ values were 8/>32 mg/L for meropenem, 8/>32 mg/L for imipenem, and 16/>32 mg/L for ertapenem.

Most isolates had high mecillinam MICs (MIC_50_ of all isolates 128 mg/L). Stratified by species lower mecillinam MICs were documented in *E. cloacae* (MIC_50/90_ 8/>256 mg/L) and *E. coli* (MIC_50/90_ 32/>256 mg/L). Stratified by carbapenemase lower mecillinam MICs were recorded in isolates producing IMI (MIC_50/90_ 8/>256 mg/L) and OXA-48-/OXA-48-like (MIC_50/90_ 16/>256 mg/L), Table 1.

**Table 1:** MICs for mecillinam and carbapenems of all isolates displayed by species and carbapenemase.

			Mecillinam mg/L			Meropenem mg/L	Imipenem mg/L	Ertapenem mg/L
			MIC 50/90	MIC median	MIC Range	MIC 50/90	MIC 50/90	MIC 50/90
*K. pneumoniae*	***n* = 47**		**256/>256**	**256**	**4->256**	**32/>32**	**>32/>32**	**32/>32**
	OXA-48-like^*^	(17)		32				
	NDM	(10)		>256				
	KPC	(9)		>256				
	VIM	(5)		>256				
	IMP	(3)		256				
	KPC + VIM	(2)		>256				
	NDM + OXA-48-like	(1)		>256				
*E. coli*	***n* = 30**		**32/>256**	**32**	**1->256**	**2/>32**	**2/>32**	**2/>32**
	OXA-48-like^*^	(16)		8				
	NDM	(7)		128				
	VIM	(4)		>256				
	NDM + OXA-48-like	(2)		256				
	IMP	(1)		64				
*E. cloacae*	***n* = 13**		**8/>256**	**8**	**2- > 256**	**>32/>32**	**>32/>32**	**>32/>32**
	IMI	(9)		8				
	VIM	(4)		>256				
*C. freundii*	***n* = 9**		**>256/>256**	**>256**	**16- > 256**	**16/>32**	**>32/>32**	**>32/>32**
	VIM	(5)		>256				
	IMP	(2)		128				
	GES	(1)		>256				
	KPC	(1)		>256				
Others	***n* = 6**		**>256**/>**256**	**>256**	**256- > 256**	**4**/**8**	**16/>32**	**1/16**
*P. mirabilis*	OXA-58	(2)		>256				
*P. mirabilis*	NDM	(1)		256				
*K. oxytoca*	VIM	(2)		>256				
*R. ornithinolytica*	NDM	(1)		>256				
**Comparators**	***n* = 3**							
*K. pneumoniae*	negative			>256		1	8	8
*E. cloacae*	negative			2		0.5	2	16
*E. coli*^ATCC^ 25922	negative			0.125				

* Including OXA-48 carbapenemases.

The group of mecillinam susceptible isolates included *E. coli* (*N* = 12), *E. cloacae* (*N* = 7), and *K. pneumoniae* (*N* = 4; Table 2). Susceptible isolates produced OXA-48 (*N* = 8), OXA-48-like (*N* = 7), IMI (*N* = 7), and NDM-1 (*N* = 1) carbapenemases. The carbapenem MICs in these isolates ranged from 0.25 mg/L to >32 mg/L. For OXA-48 producers (8/18 isolates mecillinam susceptible) and OXA-48-like producers (7/18 isolates mecillinam susceptible), low mecillinam MICs correlated with low carbapenem MICs (median MIC for imipenem 1 mg/L in mecillinam susceptible isolates; Supplementary Table S1). In IMI producers (7/9 isolates mecillinam susceptible), mecillinam was susceptible despite the high carbapenem MICs (median MIC for imipenem of >32 mg/L in mecillinam susceptible isolates). In NDM-1 producers (1/14 isolates mecillinam susceptible), median MIC for imipenem was >32 mg/L, Table 2; Supplementary Table S1.

**Table 2:** MICs for mecillinam and carbapenems of mecillinam-susceptible isolates displayed by species and carbapenemase.

Species (no. of isolates)	Carbapenemase	MIC (mg/L)				Mecillinam susceptibility rate
		Meropenem	Imipenem	Ertapenem	Mecillinam	
*K. pneumoniae* (4)	OXA-48	0.5	2	8	**4**	
	OXA-48	1	1	8	**8**	8/19 OXA-48 (42.1%) 2/3 OXA-162 (66.7%) 2/4 OXA-181 (50.0%) 1/4 OXA-232 (25.0%) 1/3 OXA-244 (33.3%) 1/2 OXA-245 (50.0%)
	OXA-162	0.5	1	2	**4**	
	OXA-245	2	2	1	**8**	
*E. coli* (12)	OXA-48	2	4	4	**8**	
	OXA-48	2	1	8	**2**	
	OXA-48	16	2	>32	**4**	
	OXA-48	2	1	>32	**8**	
	OXA-48	2	0.5	2	**8**	
	OXA-48	1	1	8	**8**	
	OXA-162	0.5	1	2	**2**	
	OXA-181	0.25	0.25	4	**8**	
	OXA-181	0.5	0.5	2	**4**	
	OXA-232	0.25	0.5	4	**8**	
	OXA-244	0.25	0.5	2	**8**	
	NDM-1	4	2	4	**1**	1/14 NDM-1 (7.1%)
*E. cloacae* (7)	IMI-1	1	>32	2	**2**	7/9 IMI (77.8%)
	IMI-2	>32	>32	>32	**8**	
	IMI-3	4	16	8	**4**	
	IMI-4	16	>32	16	**8**	
	IMI-12	>32	>32	>32	**8**	
	IMI-14	8	>32	8	**4**	
	IMI-16	>32	>32	>32	**2**	

The bold values represent the mecillinam MICs, highlighted as they are of greater significance for the interpretation of the study than the carbapenem MICs.

### Comparison of Different Methods for Susceptibility Testing

**Figure 1:**
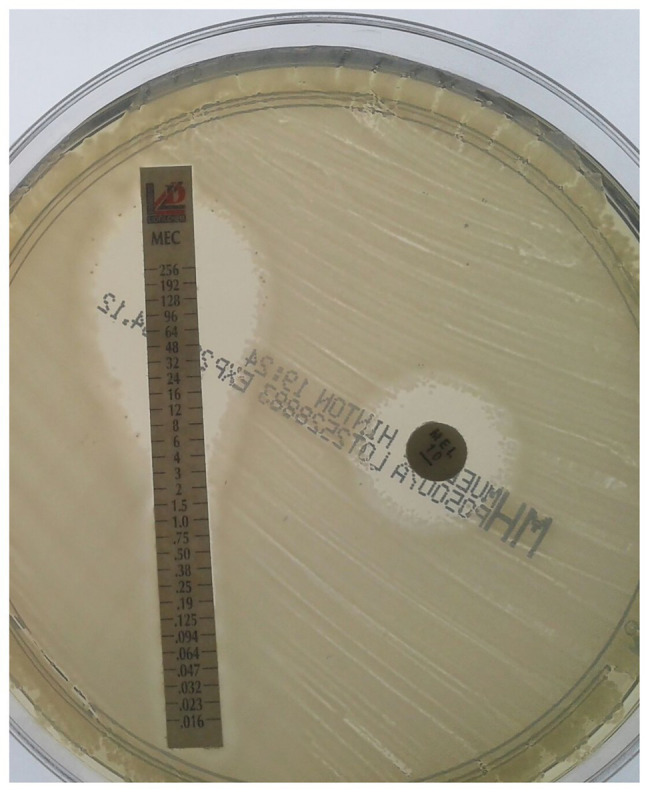
Susceptibility testing of mecillinam by agar diffusion methods in an isolate of *K. pneumoniae* producing NDM. Agar gradient diffusion and disk diffusion of the isolate with an inhibition zone diameter of 12 mm (resistant) and an MIC of 8 mg/L (susceptible) by gradient test, compared to 64 mg/L (resistant) by the reference method.

The discrimination of susceptible (≤8 mg/L) and resistant (>8 mg/L) isolates was clear when using the reference method agar dilution; borderline cases were rare in the cohort. Categorical agreement of the reference method with AGD and disk diffusion was 90.4 and 93.3%, respectively (Table 3). Compared to the high concordance of agar dilution results upon precision testing, performance of AGD and disk diffusion was more variable (Figure 1). Six isolates showed disagreement in both agar diffusion methods with agar dilution. In addition, AGD disagreed in an additional four isolates and disk diffusion in one isolate compared to agar dilution. Most major errors occurred in *K. pneumoniae* isolates (7/11; 63.6%); AGD showed more major errors (10/11) than disk diffusion (7/11). Overall AGD and disk diffusion yielded very major errors (false susceptibility) in 10/82 (12.2%) and 7/82 (8.5%) isolates, respectively. Supplementary Table S2 shows all isolates with mecillinam MICs defined as very major errors stratified by carbapenemases and MICs. No difficulties were documented in the interpretation of the carbapenemase-negative comparators, including the ATCC quality control-strain at any stage of the study.

**Table 3:** Agreement and error rates in mecillinam susceptibility testing compared to the reference method agar dilution.

	Categorical agreement^a^/all isolates (%)	Essential agreement %^b^	Very major error cases^c^/number of resistant isolates (%)	Major error cases^d^/number of susceptible isolates (%)
Agar gradient diffusion	95/105 (90.4)	71.4	10/82 (12.2)	0/23 (0.0)
Disk diffusion	98/105 (93.3)	-	07/82 (8.5)	1/23 (4.3)

a Categorical agreement was defined as the same interpretation of susceptible and resistant in each testing method.

b Essential agreement was defined as a results within +/− one doubling dilution of the MIC determined by the reference method.

c Very major error was defined as a susceptible result in agar gradient diffusion or disk diffusion while the reference method yielded a resistant result.

d Major error case was defined as a resistant result in agar gradient diffusion or disk diffusion while a susceptible result was achieved by the reference method.

## Discussion

The increasing prevalence of multidrug-resistant Enterobacterales in UTI has complicated therapy and particularly, CPE constitutes a major concern. They leave very few options for antimicrobial therapy and expedite expansion of acquired carbapenem resistance between bacteria by mobile genetic elements. Data on new or rediscovered antimicrobials which could be effective in a particular type of carbapenemase is crucial for future disease management.

To date, usage of mecillinam is established only in a few European countries (Pinart et al., 2017). In 2020, breakpoints have been updated by EUCAST to include more species, now *E. coli*, *Klebsiella* spp., *Citrobacter*, spp., *Enterobacter* spp., *Raoultella* spp., and *P. mirabilis*. Prescription of mecillinam may also rise in the future due to increasing antimicrobial resistance, introduction of mecillinam in countries outside of Europe, or the evaluation of mecillinam for other indications such as complicated UTI or bloodstream infections (Theuretzbacher et al., 2015; Jansaker et al., 2018; Fuchs et al., 2019; Boel et al., 2021). In addition, the European Medicines Agency (EMA) warning issued on the use of fluoroquinolones further limits oral therapeutic options in UTI especially in multidrug-resistant (MDR) pathogens (EMA, 2019).

In the present study, we demonstrate susceptibility of mecillinam in OXA-48-like-, IMI-, and NDM-1- producing Enterobacterales with both high and low carbapenem MICs. Compared to the results of the other groups on CRE and mecillinam, our study demonstrates that not only NDM-1 and OXA-48-like isolates show susceptibility to mecillinam but also isolates producing IMI. Susceptibility of mecillinam in IMI-producers was present despite high MICs of carbapenems (median carbapenem MIC >32 mg/L in IMI-producers), highlighting that even if isolates express their carbapenemase enzymes to a high amount, they hydrolyze mecillinam to a lesser extent compared to other beta-lactams. Since this is to the best of our knowledge the first study demonstrating mecillinam susceptibility in carbapenem resistant IMI-producers, we performed an additional agar dilution protocol with a 10 times higher inoculum in these isolates (data not shown) to minimize a confounding effect of the inoculum, which has been described previously (Thomas et al., 2006; Marrs et al., 2014). For high inoculum, testing 6/9 IMI-producers yielded mecillinam MICs of ≤8 mg/L compared to 7/9 isolates by standard methodology.

In contrast to IMI-producers, mecillinam susceptibility in OXA-48-like-producers has been previously described (Marrs et al., 2014; Mutters et al., 2015; Fuchs and Hamprecht, 2019a). For further analysis, we included *E. coli* (*N* = 18) and *K. pneumoniae* (*N* = 18) isolates with OXA-48 and different OXA-48-like carbapenemases in the challenge collection. Overall 15/36 isolates (41.7%) were mecillinam-susceptible (Table 2; Supplementary Table S1). The subgroup of OXA-48-producers (8/18 (44.4%) isolates mecillinam susceptible) consisted of nine *E. coli* and nine *K. pneumoniae* isolates, and the majority of susceptible isolates were *E. coli* (6/8; 75%). In the subgroup of OXA-48-like-producers (7/18; 38.9%) isolates mecillinam susceptible), the highest susceptibility rate was documented for OXA-162 (2/3 (66.7%) isolates mecillinam susceptible). Lower susceptibility rates were seen in OXA-181- (2/4; 50%), OXA-245- (1/2; 50%), OXA-244- (1/3; 33.3%), and OXA-232- (1/4; 25%) producers. Almost all mecillinam susceptible OXA-48 and OXA-48-like-producers had low carbapenem MICs and most of them (*N* = 11) were *E. coli* (*K. pneumoniae*, *N* = 4). Since OXA-48 and OXA-48-like are the most common carbapenemases in Western Europe and many other regions, mecillinam might be an oral alternative to carbapenem therapy for these isolates in uUTI. Previous studies reported higher rates of mecillinam susceptibility in OXA-48-like carbapenemases (up to 100% in a large Scandinavian cohort), yet these studies used agar diffusion techniques only (Samuelsen et al., 2017). Our data provide evidence for insufficient correlation of AGD and disk diffusion with the reference method agar dilution in CPE, since we found major errors in 12.2 and 8.5%, respectively. Another study that used the reference methodology agar dilution yielded a mecillinam susceptibility of 21.4% in 3/14 clinical isolates producing OXA-48-like carbapenemases (Marrs et al., 2014). To conclude, it should be considered that mecillinam susceptibility in CPE may be overestimated if agar diffusion techniques only are used for testing.

Compared to previous studies (Perry et al., 2011; Marrs et al., 2014), mecillinam susceptibility in NDM producers was low in our cohort. These studies yielded susceptibility rates for NDM-1 producers of 95% and an MIC_90_ of 8 mg/L. The isolates in these cohorts were almost exclusively from Pakistan, included NDM-1 only (*N* = 64) and were in the majority *E. coli* (*N* = 30) or *E. cloacae* (*N* = 21) with low carbapenem MICs. The authors state that the susceptibility rate is high for NDM-1 especially in *E. coli* and *E. cloacae* in contrast to *K. pneumoniae*. On the contrary, our cohort included different types of NDM-producers [NDM-1 (*N* = 14), NDM-5 (*N* = 2), NDM-9 (*N* = 2), NDM-3 (*N* = 1), NDM-4 (*N* = 1), NDM-7 (*N* = 1), and NDM-8 (*N* = 1); Supplementary Table S1] and *K. pneumoniae* was the most frequent species (*N* = 11). Of four NDM-1 producing *E. coli*, only one was susceptible yet the other three had either high carbapenem MICs or produced a second carbapenemase gene (Table 2; Supplementary Table S1). Mecillinam susceptibility in NDM-producing Enterobacterales might therefore be limited to the NDM-1-subtype and restricted to some species and/or isolates with low carbapenem MICs. Of note, *in vivo* success of mecillinam was documented in a murine UTI-model with one NDM-1 producing *E. coli* with low carbapenem MICs (Zykov et al., 2020).

In line with other studies, poor mecillinam activity was documented in KPC-, IMP, and VIM- producers, irrespective of species (Marrs et al., 2014). Data on *P. mirabilis* as multidrug-resistant pathogen are limited, our study included three isolates [OXA-58 (*N* = 2) and NDM-1 (*N* = 1)], all of which had a mecillinam MIC of ≥256 mg/L.

Our study has some limitations. Isolates in this study were not only from urine but from different clinical specimens. Species other than *E. coli*, which causes most uUTI, were over-represented. Nevertheless, all CPE in the challenge collection have been reported as the cause of UTI, and we included a large variety of molecularly characterized CPE. However, for some rare carbapenemases only few isolates could be assessed (e.g., GES), and generalizability is limited for these variants. Furthermore, some isolates in our collection had low carbapenem MICs, indicating a low expression of the detected carbapenemase enzymes. Besides the level of gene expression, clinical isolates carrying carbapenemases can additionally have other mechanisms that might impact the susceptibility against mecillinam such as previous antimicrobial therapy or other beta-lactamases or porin modifications, which were not the focus of our study.

In general, *in vivo* data on the potential of mecillinam to achieve microbiological cure are still scarce in other species than *E. coli*, and treatment failure despite mecillinam susceptibility has been documented in UTI (Søraas et al., 2014). Especially for infections caused by CPE data on clinical outcome after mecillinam therapy is not available, and low MICs do not necessarily correlate with clinical success. Nevertheless, mecillinam might be considered for therapy in uUTI caused by *E. coli* and *E. cloacae* isolates carrying IMI-, OXA-48, and OXA-48-like- carbapenemases. Future studies are warranted on *in vivo* activity of mecillinam against infections caused by CPE.

To conclude, with the increasing prevalence of MDR pathogens in inpatient and outpatient UTI, oral treatment has become more and more difficult. In this large cohort of CPE, we showed that mecillinam susceptibility is restricted to isolates producing the carbapenemases IMI, OXA-48-like, and NDM-1 and is more frequent in *E. coli* and *E. cloacae* than in other species. Even in isolates with high carbapenem MICs, mecillinam can still be active. Further research is needed on appropriate susceptibility testing in clinical routine and the correlation of mecillinam therapy and clinical outcome in CPE infections.

## Data Availability Statement

The original contributions presented in the study are included in the article/Supplementary Material, further inquiries can be directed to the corresponding author.

## Author Contributions

AH: conceptualization, supervision, validation, resources, and writing-review and editing. FF: project administration, investigation, writing-original draft, and writing-review and editing. AA, LP, and TW: Investigation. All authors contributed to the article and approved the submitted version.

### Conflict of Interest

The authors declare that the research was conducted in the absence of any commercial or financial relationships that could be construed as a potential conflict of interest.

## References

[ref1] Baeza L. L. Pfennigwerth N. Greissl C. Göttig S. Saleh A. Stelzer Y.. (2019). Comparison of five methods for detection of carbapenemases in Enterobacterales with proposal of a new algorithm. Clin. Microbiol. Infect. 25, 1286.e9–1286.e15. 10.1016/j.cmi.2019.03.003, PMID: 30898725

[ref2] Boel J. B. Antsupova V. Knudsen J. D. Jarløv J. O. Arpi M. Holzknecht B. J. (2021). Intravenous mecillinam compared with other β-lactams as targeted treatment for *Escherichia coli* or Klebsiella spp. bacteraemia with urinary tract focus. J. Antimicrob. Chemother. 76, 206–211. 10.1093/jac/dkaa411, PMID: [Epub ahead of print] 32989447

[ref3] CLSI (2020). Performance standards for antimicrobial susceptibility testing. *30th Edn.* CLSI supplement M100. Wayne: Clinical and Laboratory Standards Institute.

[ref4] EMA (2019). Disabling and Potentially Permanent Side Effects Lead to Suspension or Restrictions of Quinolone and Fluoroquinolone Antibiotics. Amsterdam; 1–4. Available at: https://www.ema.europa.eu/en/news/disabling-potentially-permanent-side-effects-lead-suspension-restrictions-quinolone-fluoroquinolone (Accessed November 11, 2020).

[ref5] EUCAST (2000). Determination of minimum inhibitory concentrations (MICs) of antibacterial agents by agar dilution. Clin. Microbiol. Infect. 6, 509–515. 10.1046/j.1469-0691.2000.00142.x 11168187

[ref6] EUCAST (2018). Routine and extended internal quality control for MIC determination and disk diffusion as recommended by EUCAST. Version 8.0.

[ref7] EUCAST (2020). Breakpoint tables for interpretation of MICs and zone diameters. Version 10.0. Available at: http://www.eucast.org (Accessed January 20, 2020).

[ref8] Fuchs F. Hamprecht A. (2019a). Results from a prospective in vitro study on the mecillinam (amdinocillin) susceptibility of Enterobacterales. Antimicrob. Agents Chemother. 63, e02402–e02418. 10.1128/AAC.02402-18, PMID: 30917983 PMC6437539

[ref9] Fuchs F. Hamprecht A. (2019b). Susceptibility of carbapenemase-producing Enterobacterales (CPE) to nitroxoline. J. Antimicrob. Chemother. 74, 2934–2937. 10.1093/jac/dkz275, PMID: 31292653

[ref10] Fuchs F. Wille J. Hamprecht A. Parcina M. Lehmann C. Schwarze-Zander C.. (2019). In vitro activity of mecillinam and nitroxoline against *Neisseria gonorrhoeae* – re-purposing old antibiotics in the multi-drug resistance era. J. Med. Microbiol. 68, 991–995. 10.1099/jmm.0.001014, PMID: 31162022

[ref11] Greissl C. Saleh A. Hamprecht A. (2019). Rapid detection of OXA-48-like, KPC, NDM, and VIM carbapenemases in Enterobacterales by a new multiplex immunochromatographic test. Eur. J. Clin. Microbiol. Infect. Dis. 38, 331–335. 10.1007/s10096-018-3432-2, PMID: 30448931

[ref12] Gruber T. M. Gottig S. Mark L. Christ S. Kempf V. A. J. Wichelhaus T. A.. (2015). Pathogenicity of pan-drug-resistant *Serratia marcescens* harbouring blaNDM-1. J. Antimicrob. Chemother. 70, 1026–1030. 10.1093/jac/dku482, PMID: 25468904

[ref13] Gupta K. Hooton T. M. Naber K. G. Wullt B. Colgan R. Miller L. G.. (2011). International clinical practice guidelines for the treatment of acute uncomplicated cystitis and pyelonephritis in women: a 2010 update by the Infectious Diseases Society of America and the European Society for Microbiology and Infectious Diseases. Clin. Infect. Dis. 52, e103–e120. 10.1093/cid/ciq257, PMID: 21292654

[ref14] Hamprecht A. Vehreschild J. J. Seifert H. Saleh A. (2018). Rapid detection of NDM, KPC and OXA-48 carbapenemases directly from positive blood cultures using a new multiplex immunochromatographic assay. PLoS One 13:e0204157. 10.1371/journal.pone.0204157, PMID: 30216371 PMC6138386

[ref15] Jansaker F. Frimodt-Moller N. Benfield T. L. Knudsen J. D. (2018). Mecillinam for the treatment of acute pyelonephritis and bacteremia caused by Enterobacteriaceae: a literature review. Infect. Drug Resist. 11, 761–771. 10.2147/IDR.S163280, PMID: 29872326 PMC5973435

[ref16] Koroska F. Goettig S. Kaase M. Steinmann J. Gatermann S. G. Sommer J.. (2017). Comparison of phenotypic tests and an mmunochromatographic assay and development of a new algorithm for detection of OXA-48-like carbapenemases. J. Clin. Microbiol. 55, 877–883. 10.1128/JCM.01929-16, PMID: 28031433 PMC5328455

[ref17] Kranz J. Schmidt S. Lebert C. Schneidewind L. Vahlensieck W. Sester U.. (2017). Epidemiology, diagnostics, therapy, prevention and management of uncomplicated bacterial outpatient acquired urinary tract infections in adult patients: update 2017 of the interdisciplinary AWMF S3 guideline. Urologe A 56, 746–758. 10.1007/s00120-017-0389-1, PMID: 28455578

[ref18] Lund F. Tybring L. (1972). 6-amidinopenicillanic acids - a new group of antibiotics. Nat. New Biol. 236, 135–137. 4402006 10.1038/newbio236135a0

[ref19] Marrs E. C. Day K. M. Perry J. D. (2014). In vitro activity of mecillinam against Enterobacteriaceae with NDM-1 carbapenemase. J. Antimicrob. Chemother. 69, 2873–2875. 10.1093/jac/dku204, PMID: 24917584

[ref20] Martin A. Fahrbach K. Zhao Q. Lodise T. (2018). Association between carbapenem resistance and mortality among adult, hospitalized patients with serious infections due to Enterobacteriaceae: results of a systematic literaturereview and meta-analysis. Open Forum Infect. Dis. 5:ofy150. 10.1093/ofid/ofy150, PMID: 30046639 PMC6054228

[ref21] Matuschek E. Brown D. F. Kahlmeter G. (2014). Development of the EUCAST disk diffusion antimicrobial susceptibility testing method and its implementation in routine microbiology laboratories. Clin. Microbiol. Infect. 20, O255–O266. 10.1111/1469-0691.12373, PMID: 24131428

[ref22] Mazzariol A. Bazaj A. Cornaglia G. (2017). Multidrug-resistant gram-negative bacteria causing urinary tract infections: a review. J. Chemother. 29, 2–9. 10.1080/1120009X.2017.1380395 29271736

[ref23] Mutters N. T. Zimmermann S. Kaase M. Mischnik A. (2015). Activity of temocillin, mecillinam, ceftazidime, and ceftazidime/avibactam against carbapenem-non-susceptible Enterobacteriaceae without carbapenemase production. Eur. J. Clin. Microbiol. Infect. Dis. 34, 2429–2437. 10.1007/s10096-015-2498-3, PMID: 26433746

[ref24] Neu J. C. (1976). Synergy of mecillinam, a beta-amidinopenicillanic acid derivative, combined with beta-lactam antibiotics. Antimicrob. Agents Chemother. 10, 535–542. 984795 10.1128/aac.10.3.535 PMC429784

[ref25] Nordmann P. Poirel L. (2014). The difficult-to-control spread of carbapenemase producers among Enterobacteriaceae worldwide. Clin. Microbiol. Infect. 20, 821–830. 10.1111/1469-0691.12719, PMID: 24930781

[ref26] Perry J. D. Naqvi S. H. Mirza I. A. Alizai S. A. Hussain A. Ghirardi S.. (2011). Prevalence of faecal carriage of Enterobacteriaceae with NDM-1 carbapenemase at militaryhospital in Pakistan, and evaluation of two chromogenic media. J. Antimicrob. Chemother. 66, 2288–2294. 10.1093/jac/dkr299, PMID: 21788293

[ref27] Pinart M. Kranz J. Jensen K. Proctor T. Naber K. Kunath F.. (2017). Optimal dosage and duration of pivmecillinam treatment for uncomplicated lower uri-nary tract infections: a systematic review and meta-analysis. Int. J. Infect. Dis. 58, 96–109. 10.1016/j.ijid.2017.03.012, PMID: 28341436

[ref28] Samuelsen Ø. Overballe-Petersen S. Bjørnholt J. V. Brisse S. Doumith M. Woodford N.. (2017). Molecular and epidemiological characterization of carbapenemase-producing Enterobacteriaceae in Norway, 2007 to 2014. PLoS One 12:e0187832. 10.1371/journal.pone.0187832, PMID: 29141051 PMC5687771

[ref29] Søraas A. Sundsfjord A. Jørgensen S. B. Liestøl K. Jenum P. A. (2014). High rate of per oral mecillinam treatment failure in community-acquired urinary tract infections caused by ESBL-producing *Escherichia coli*. PLoS One 9:e85889. 10.1371/journal.pone.0085889, PMID: 24454943 PMC3893261

[ref30] Theuretzbacher U. Van Bambeke F. Canton R. Giske C. G. Mouton J. W. Nation R. L.. (2015). Reviving old antibiotics. J. Antimicrob. Chemother. 70, 2177–2181. 10.1093/jac/dkv157, PMID: 26063727

[ref31] Thomas K. Weinbren M. J. Warner M. Woodford N. Livermore D. (2006). Activity of mecillinam against ESBL producers in vitro. J. Antimicrob. Chemother. 57, 367–368. 10.1093/jac/dki451, PMID: 16354745

[ref32] Zilberberg M. D. Nathanson B. H. Sulham K. Fan W. Shorr A. F. (2017). Carbapenem resistance, inappropriate empiric treatment and outcomes among patients hospitalized with Enterobacteriaceae urinary tract infection, pneumonia and sepsis. BMC Infect. Dis. 17:279. 10.1186/s12879-017-2383-z, PMID: 28415969 PMC5393012

[ref33] Zykov I. N. Frimodt-Møller N. Småbrekke L. Sundsfjord A. Samuelsen Ø. (2020). Efficacy of mecillinam against clinical multidrug-resistant *Escherichia coli* in a murine urinary tract infection model. Int. J. Antimicrob. Agents 55:105851. 10.1016/j.ijantimicag.2019.11.008, PMID: 31770624

