# Supplementary material for: Susceptibility of Clinical Enterobacterales Isolates With Common and Rare Carbapenemases to Mecillinam
Source: Front Microbiol. 2021 Jan 12;11:627267. doi: 10.3389/fmicb.2020.627267 (PMC7835630; doi:10.3389/fmicb.2020.627267)
Supplement: Supplementary file 1 [file Data_Sheet_1.pdf]

Susceptibility of clinical Enterobacterales isolates with common and rare carbapenemases to mecillinam

Frieder Fuchs[1], Aysel Ahmadzada[1], Lars Plambeck[1], Thorsten Wille[1] and Axel Hamprecht[1,2,3*]

[1]Institute for Medical Microbiology, Immunology and Hygiene, University of Cologne, Medical faculty and University Hospital of Cologne, Cologne, Germany

[2]German Centre for Infection Research (DZIF), partner site Bonn-Cologne, Cologne, Germany

[3]Institute for Medical Microbiology and Virology, University of Oldenburg, Germany

*** Correspondence:**
Axel Hamprecht (corresponding author), University of Cologne, Institute for Medical Microbiology, Immunology and Hygiene, Cologne, Germany
Telephone: +49 221-478 32009
e-mail: axel.hamprecht@uk-koeln.de

Supplementary data

Table S1 Isolates displayed by MIC, species and carbapenemase.
Mecillinam breakpoint S ≤ 8 mg/L, R > 8 mg/L (EUCAST)

**Species (no. of isolates)**	**Carbapenemase**	**MIC (mg/L)**			
		Meropenem	Imipenem	Ertapenem	Mecillinam
**IMI**					
E. cloacae (9)	IMI-1	1	>32	2	2
	IMI-2	>32	>32	>32	8
	IMI-3	4	16	8	4
	IMI-4	16	>32	16	8
	IMI-9	16	>32	>32	16
	IMI-10	>32	>32	>32	>256
	IMI-12	>32	>32	>32	8
	IMI-14	8	>32	8	4
	IMI-16	>32	>32	>32	2
**NDM**					
K. pneumonia (10)	NDM-1	8	8	8	32
	NDM-1	8	4	>32	32
	NDM-1	32	16	16	>256
	NDM-1	32	>32	>32	128
	NDM-1	>32	>32	>32	16
	NDM-1	>32	>32	>32	>256
	NDM-1	>32	>32	>32	>256
	NDM-8	32	32	>32	64
	NDM-9	4	8	8	16
	NDM-9	4	4	8	128
E. coli (7)	NDM-1	4	2	4	1
	NDM-1	>32	>32	>32	>256
	NDM-1	>32	>32	>32	>256
	NDM-3	2	4	4	32
	NDM-4	32	16	32	64
	NDM-5	>32	>32	>32	32
	NDM-7	>32	>32	>32	128
R. ornithinolytica (1)	NDM-1	8	8	16	64
P. mirabilis (1)	NDM-1	1	8	0.125	256

Species (no. of isolates)	Carbapenemase	MIC (mg/L)			
		Meropenem	Imipenem	Ertapenem	Mecillinam
**OXA**					
K. pneumoniae (17)	OXA-48	16	4	32	64
	OXA-48	32	16	8	128
	OXA-48	2	4	2	256
	OXA-48	1	2	>32	16
	OXA-48	2	2	4	16
	OXA-48	0.5	2	8	4
	OXA-48	1	0.5	8	8
	OXA-48	16	8	32	32
	OXA-48	2	2	32	256
	OXA-162	0.5	1	2	4
	OXA-162	32	6	32	16
	OXA-204	32	8	32	64
	OXA-232	>32	>32	>32	128
	OXA-244	32	32	32	>256
	OXA-245	32	32	4	256
	OXA-245	2	2	1	8
	OXA-370	4	4	16	16
E. coli (16)	OXA-48	2	4	4	8
	OXA-48	2	1	8	2
	OXA-48	16	2	>32	4
	OXA-48	2	0.5	2	8
	OXA-48	0.25	2	1	16
	OXA-48	2	1	>32	8
	OXA-48	1	1	8	8
	OXA-48	8	4	8	>256
	OXA-162	0.5	1	2	2
	OXA-181	0.25	0.5	2	16
	OXA-181	0.5	0.5	2	4
	OXA-181	0.25	0.25	2	8
	OXA-232	0.25	0.5	2	>256
	OXA-232	0.25	0.5	4	8
	OXA-244	0.25	0.5	2	8
	OXA-244	4	4	2	64
P. mirabilis (2)	OXA-58	16	>32	1	>256
	OXA-58	4	>32	2	>256

Species (no. of isolates)	Carbapenemase	MIC (mg/L)			
		Meropenem	Imipenem	Ertapenem	Mecillinam
**VIM**					
Klebsiella spp. (7)	VIM-1	4	8	0.5	>256
	VIM-1	4	4	1	>256
	VIM-2	0.125	1	0.06	>256
	VIM-2	0.25	0.5	0.125	>256
	VIM-4	2	16	1	>256
	VIM-46	0.5	4	0.5	>256
	VIM-52	8	>32	32	>256
C. freundii (5)	VIM-2	0.25	0.5	1	>256
	VIM-5	2	4	4	256
	VIM-31	0.5	1	0.5	>256
	VIM-51	>32	>32	>32	>256
	VIM-59	>32	>32	>32	>256
E. cloacae (4)	VIM-4	1	1	8	>256
	VIM-26	>32	>32	>32	>256
	VIM-27	>32	>32	>32	>256
	VIM-39	>32	>32	>32	>256
E. coli (4)	VIM-1	0.5	4	0.25	>256
	VIM-1	16	>32	0.5	>256
	VIM-1	2	4	0.06	>256
	VIM-1	0.125	0.5	0.125	>256
**IMP**					
K. pneumoniae (3)	IMP-1	16	8	>32	256
	IMP-4	4	1	4	256
	IMP-22	>32	>32	>32	128
C. freundii (2)	IMP-1	2	1	2	16
	IMP-8	16	>32	>32	128
E. coli (1)	IMP-14	0.5	0.25	0.5	64
**KPC**					
K. pneumoniae (9)	KPC-2	>32	>32	>32	>256
	KPC-2	>32	32	16	>256
	KPC-2	>32	>32	>32	>256
	KPC-2	>32	>32	>32	>256
	KPC-2	32	32	32	>256
	KPC-2	>32	>32	>32	>256
	KPC-2	>32	>32	>32	>256
	KPC-2	>32	>32	>32	>256
	KPC-3	>32	>32	>32	>256
C. freundii (1)	KPC-3	>32	>32	>32	>256
**GES**					
C. freundii (1)	GES-25	>32	>32	>32	>256

**Isolates with two carbapenemases**					
K. pneumoniae (3)	KPC-2+VIM-1	>32	>32	>32	>256
	KPC-2+VIM-1	>32	>32	>32	>256
	NDM-1+OXA-232	>32	>32	>32	>256
E. coli (2)	NDM-5+OXA-181	>32	>32	8	64
	NDM-1+OXA-48	0.125	0.125	2	>256

Table S2 Isolates with very major errors by agar gradient diffusion or disk diffusion. Each isolate was assessed at least 3 times, with the same inoculum used for all techniques. Mecillinam breakpoint S ≤ 8 mg/L, R > 8 mg/L (EUCAST)

**Species (no. of isolates)**	**Carbapenemase**	**Susceptibility-Testing of mecillinam**			
		Disk diffusion	Agar gradient diffusion	Agar dilution	Mecillinam MIC (mg/L)
K. pneumoniae (7)	NDM-1	S	S	R	32
	NDM-8	R	S	R	64
	NDM-9	S	S	R	16
	OXA-370	S	S	R	16
	OXA-245	S	R	R	256
	VIM-2	R	S	R	>256
	IMP-1	R	S	R	256
E. coli (4)	OXA-48	S	S	R	16
	OXA-181	S	S	R	16
	NDM-3	S	S	R	32
	IMP-14	R	S	R	64
